# Incidental Meckel’s Diverticulum in Umbilical Hernia

**DOI:** 10.7759/cureus.41115

**Published:** 2023-06-28

**Authors:** Sagar Prakash, Julia Sunil, Madan Shivakumar, Oseen Shaikh, Gopal Balasubramanian

**Affiliations:** 1 Surgery, Jawaharlal Institute of Postgraduate Medical Education and Research, Puducherry, IND

**Keywords:** hernia, meshplasty, littre's hernia, umbilical hernia, meckel's diverticulum

## Abstract

The presence of a Meckel's diverticulum in the hernial sac is called a Littre's hernia. It is a rare complication of Meckel's diverticulum. We present a 56-year-old male patient who complained of swelling in the umbilical region. After the clinical examination and imaging studies, we diagnosed the patient with a partially reducible umbilical hernia. The patient underwent exploration and was found to have omentum, Meckel's diverticulum, and part of the ileum within the sac. The patient underwent segmental resection of the bowel, primary repair of the hernial defect, and onlay meshplasty. Postoperatively, the patient did not develop any complications.

## Introduction

Meckel's diverticulum (MD), being present in about 2% of the adult population, is one of the commonest congenital anomalies of the gastrointestinal tract. MD has a complication rate of 4-6% [1]. It can develop complications like gastrointestinal bleeding, bowel obstruction, inflammation, and perforation. Gastrointestinal bleeding is the most common complication, followed by small bowel obstruction. Although rare, MD can present as content within the hernial sac, called Littre's hernia (LH). The existence of MD in a hernia sac is quite rare, and its exact frequency remains unknown [1]. It can occur at the inguinal, femoral, or umbilicus [1-3]. Inguinal is the most common site for LH, and umbilical is rare comparatively. LH may develop complications like MD, including intestinal obstruction, perforation, or incarceration [4]. Preoperative imaging is not helpful as it does not diagnose LH definitively [1]. These are usually diagnosed intraoperatively, and preoperative diagnosis is difficult. Treatment of LH includes treatment of MD and repair of the hernial defect. MD can be managed by diverticulectomy or segmental resection of the bowel. Risk factors like age of fewer than 40 years, having a band attached to MD, presence of heterotopic mucosa, and having long MD with a narrow base, can increase the risk of MD complications in the future and should undergo segmental resection. Mesh is routinely used in patients whose hernial defect is large, and there is no evidence of contamination. The mesh is usually not used in the presence of contamination like incarceration or perforation. In an earlier study, a mesh was used in 17% of the patients when there was the presence of incarceration or perforation [1]. This report describes the case of a 56-year-old male patient who presented to our outpatient department with swelling at the umbilical region and was diagnosed with a partially reducible umbilical hernia. Intraoperatively, surgeons noticed the presence of an MD within the hernial sac. The patient was managed by segmental resection of the bowel and onlay meshplasty.

## Case presentation

**Figure 1:**
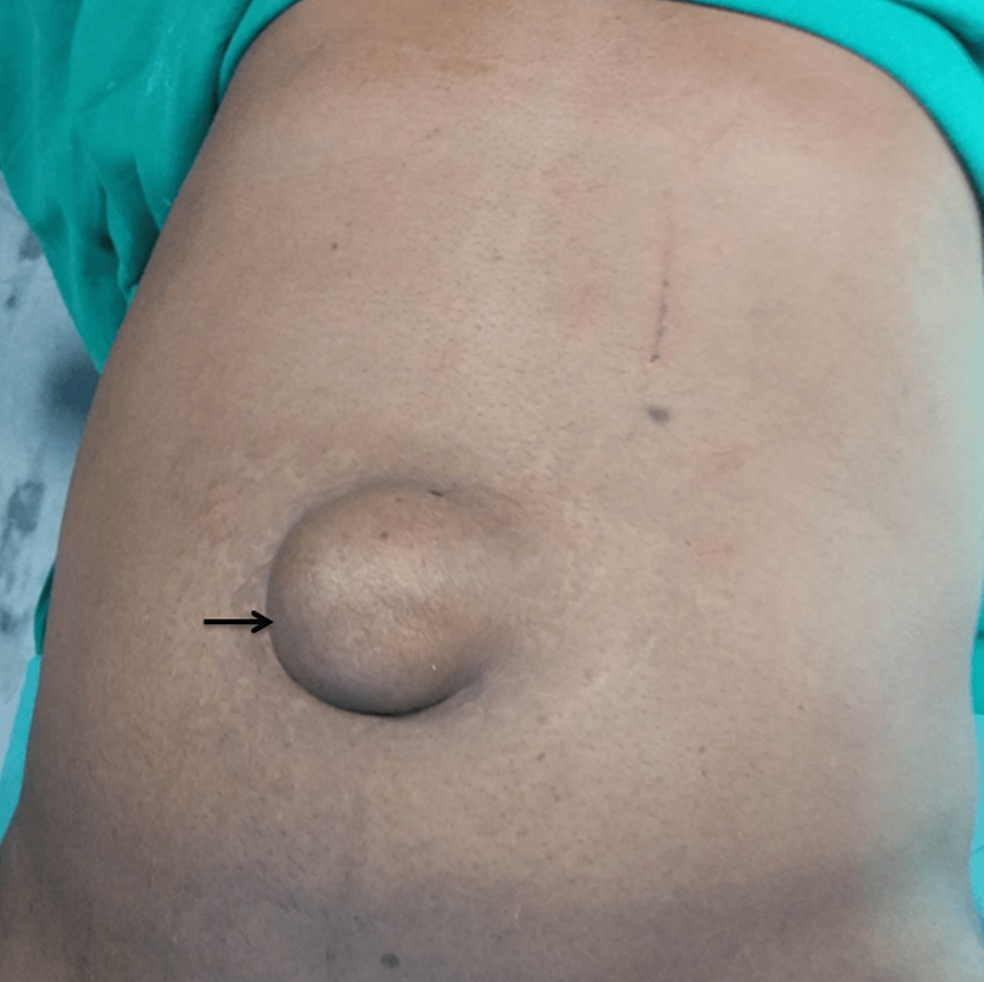
Preoperative image showing umbilical hernia (arrow).

A 56-year-old male presented to the outpatient department with a history of protrusion at the umbilical region since childhood. Initially, the swelling was small and remained unchanged until age 40. However, over the past 15 years, it had progressively increased in size. The swelling did not reduce in the lying down position. The patient complained of mild, dull aching pain over the swelling for the last two weeks. There was no history of pain associated with swelling, severe abdomen pain, nausea, vomiting, constipation, or abdominal distention. On examination, there was a large swelling over the umbilical region, measuring almost 6 cm x 6 cm, firm, and only partially reducible (Figure 1).

There was a mild cough impulse present over the swelling. The skin over the swelling was mildly stretched, and there was no evidence of ascites or any features of cirrhosis. Inguinal and femoral hernial orifices were normal. 

Routine blood investigations like hemoglobin, white blood cell (WBC) count, renal function test (RFT), and liver function test (LFT) were normal. The abdominal X-ray did not show any evidence of obstruction. Ultrasonography (USG) of the abdomen showed the presence of a 3 cm hernia defect at the umbilicus containing bowel and omentum as content. Considering all this, we diagnosed the patient with a partially reducible umbilical hernia. The patient was planned for umbilical hernia repair with meshplasty.

**Figure 2:**
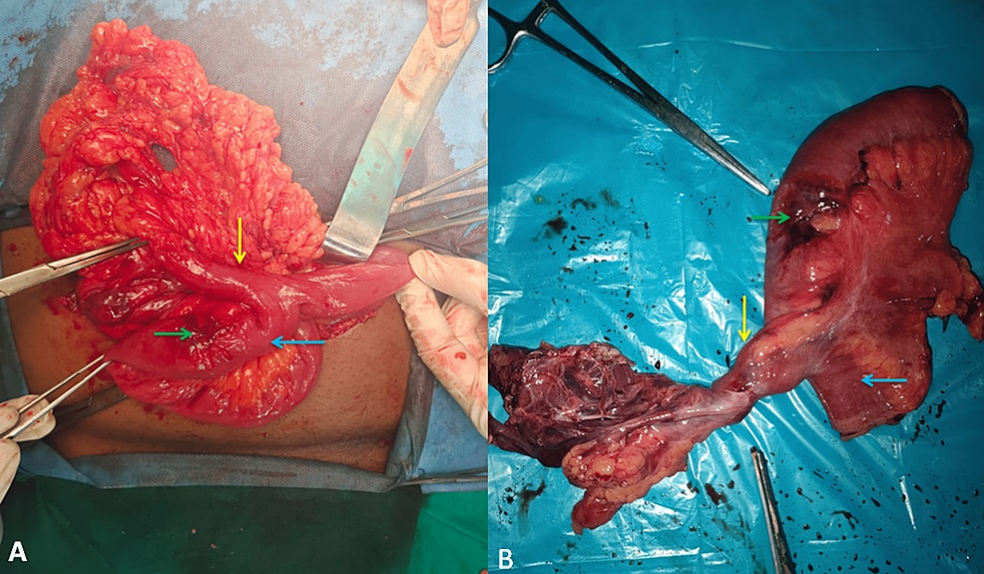
(A) Intraoperative image and (B) Resected specimen showing small bowel loop (blue arrow), Meckel’s diverticulum (yellow arrow), and focal thickening in the bowel wall near the base of the diverticulum (green arrow)

The patient underwent exploration under general anesthesia and was found to have an omentum, MD, and part of the small bowel loop as the content of the umbilical hernia. The MD was long, almost, measuring 4 cm to 5 cm, with a narrow base arising from the antimesenteric border of the terminal ileum. It was about two feet from the ileocecal junction, with a thickening near the base of the MD about 1 cm away from it. The apex of the MD had a fibrous band attached to the hernial sac. Considering three predisposing factors that may increase the risk of MD complications in the future, segmental resection of the bowel containing diverticulum was done (Figure 2). These factors were long MD with a narrow base, attachment of the fibrous band at the apex, and the possibility of ectopic mucosa, evidenced by adjoining thickening of the bowel.

After the segmental resection of the bowel, surgeons did manual, end-to-end, isoperistaltic anastomosis with Vicryl 3-0 suture (Ethicon Inc., Raritan, New Jersey, United States) in two layers. We closed the hernial defect primarily with polydioxanone 1-0 suture in a continuous fashion. Onlay meshplasty was done using Prolene mesh (15 cm x 15 cm) (Ethicon Inc.) and fixed with Prolene 2-0 suture (Ethicon Inc.). The patient was discharged after a week without any complications.

The patient was advised to follow up weekly in the outpatient department for the next two weeks. During this period, the patient had no complaints of surgical site pain or bowel habit changes. The histopathology of the resected specimen was consistent with MD without any evidence of the ectopic mucosa. The patient was regularly followed up for the next six months, and there were no symptoms of recurrence of the swelling.

## Discussion

LH is usually present as an inguinal, umbilical, femoral hernia, obturator, Spigelian, and ventral abdominal wall hernia [1-3]. LH has been categorized into two types: (i) "True LH", which contains only MD, and (ii) "Mixed LH", which contains MD and other intraabdominal organs like the small bowel [1]. Our patient had mixed LH, intraoperatively found to have MD, omentum, and ileal loop. LH is more commonly seen in females than males [5]. They proposed that the probable reason for LH to be common in females is due to the high incidence of femoral and obturator hernias. LH at the inguinal and Spigelian regions is more common in males, and the rest of the sites are more common in females [5]. Although umbilical LH is more common in females, our patient was male.

LH may present like a usual hernia, which may be inguinal, femoral, or umbilical. LH is usually diagnosed intraoperatively, and difficult to comment on the presence of LH preoperatively. LH may present with complications like MD, but these are uncommon. It may be strangulated, incarcerated, or perforated [4]. Although very rare, LH may be incarcerated without any evidence of obstruction. In such cases, only the MD is present in the hernia sac, without any other part of the bowel, just like a Richter hernia [6]. It is extremely rare to see an entero-cutaneous fistula from the MD in LH at the umbilicus [7]. A case report in the literature shows that the patient with LH at the umbilicus had incarcerated and strangulated omentum with normal MD [8]. Our patient had partially reducible, mixed LH at the umbilicus without signs of obstruction, incarceration, or perforation.

Diagnosis of LH preoperatively is difficult and is usually done intraoperatively. A plain radiograph may be helpful if there are features of intestinal obstruction. However, it cannot diagnose the cause of it. Abdominal USG and computed tomography (CT) are helpful but cannot diagnose definitively [1]. Nuclear imaging has also been used to diagnose MD; however, the sensitivity and specificity in adults are low. Single photon emission computed tomography (SPECT) scans with a CT have been used for diagnosing MD. Few pharmacological agents are used to increase the sensitivities of nuclear imaging. However, their role in LH diagnosis is doubtful [9]. In our patient, we did not suspect LH; only USG abdomen was done, which showed no evidence of LH.

The treatment of LH includes the management of MD and hernial defects. Patients with complicated LH where the MD is perforated, incarcerated, or has a bowel obstruction must undergo MD resection and repair of the hernial defect. For patients who have LH and are diagnosed incidentally during the repair of inguinal, femoral, or umbilical hernia, the MD can be left as it is, and hernial defect only can be repaired. There are a few indications where the MD has to be resected, even if found incidentally. These include patients who are less than 40 years of age, have a band attached to MD, have the presence of heterotopic mucosa, and have long MD with a narrow base [8]. The presence of malignant tumors within the MD will require wide bowel and mesentery resection [8]. MD can be resected by doing diverticulectomy or segmental bowel resection. If the base is narrow, diverticulectomy can be done, followed by horizontal closure of the ileum. If the base of the MD is wide, or if there is bleeding perforation or presence of the heterotopic mucosa, these should undergo segmental resection of the bowel. This is because of the possibility of ulcerated adjacent ileum, which may cause recurrent bleeding. Surgeries incorporate any potential ileal ulceration within the resection specimen by performing resection.

The second essential component of LH is the repair of the hernial defect. All patients should undergo closure of the hernia defect. Controversy exists regarding the usage of mesh reinforcement for the repair and reinforcement of the hernial defect. However, mesh has been used in a few patients with incarceration or perforation [1]. Ideal mesh placement should be retromuscular or preperitoneal. In the described case, we did onlay meshplasty, as we had minimal controlled contamination due to segmental resection, which might reduce the risk of infection. 

## Conclusions

MD in the umbilical hernia, also called LH, is a rare complication. LH may be complicated by bowel obstruction, incarceration, or perforation. They are found incidentally also within the hernia sac. Perhaps asymptomatic MD can be left alone. However, if the patient is young, ectopic tissue is present, a band is present, or MD is long and narrow, then it has to be resected. Hernia defect also has to be treated for prevention of the recurrence of the hernia. 
